# Anthropogenic electromagnetic fields (EMF) influence the behaviour of bottom-dwelling marine species

**DOI:** 10.1038/s41598-020-60793-x

**Published:** 2020-03-06

**Authors:** Zoë L. Hutchison, Andrew B. Gill, Peter Sigray, Haibo He, John W. King

**Affiliations:** 1 Graduate School of Oceanography, University of Rhode Island, South Kingstown, USA; 2 PANGALIA Environmental, Bedfordshire, England UK; 3 Cefas, Centre for Environment, Fisheries and Aquaculture Science, Suffolk, England UK; 4 FOI, Department of Underwater Research, Stockholm, Sweden; 5 Department of Electrical, Computer and Biomedical Engineering, University of Rhode Island, South Kingstown, USA

**Keywords:** Behavioural ecology, Behavioural ecology, Environmental impact, Marine biology, Marine biology

## Abstract

Many marine animals have evolved sensory abilities to use electric and magnetic cues in essential aspects of life history, such as to detect prey, predators and mates as well as to orientate and migrate. Potential disruption of vital cues by human activities must be understood in order to mitigate potential negative influences. Cable deployments in coastal waters are increasing worldwide, in capacity and number, owing to growing demands for electrical power and telecommunications. Increasingly, the local electromagnetic environment used by electro- and magneto-sensitive species will be altered. We quantified biologically relevant behavioural responses of the presumed, magneto-receptive American lobster and the electro-sensitive Little skate to electromagnetic field (EMF) emissions of a subsea high voltage direct current (HVDC) transmission cable for domestic electricity supply. We demonstrate a striking increase in exploratory/foraging behaviour in skates in response to EMF and a more subtle exploratory response in lobsters. In addition, by directly measuring both the magnetic and electric field components of the EMF emitted by HVDC cables we found that there were DC and unexpectedly AC components. Modelling, restricted to the DC component, showed good agreement with measured results. Our cross-disciplinary study highlights the need to integrate an understanding of the natural and anthropogenic EMF environment together with the responses of sensitive animals when planning future cable deployments and predicting their environmental effects.

## Introduction

Electromagnetic fields (EMFs) pervade the whole of the earth’s environment and have been present throughout evolution of life on earth. The most dominant natural EMFs in the marine environment are the Earth’s geomagnetic field (25–65 µT) and motionally induced electric fields, resulting from conductive seawater moving through the geomagnetic field^1^. Organisms themselves also emit important but weak bioelectric fields resulting from cellular processes and muscular movements^2^.

Electromagnetic (EM) senses in marine animals have evolved multiple times across many taxa with a variety of, and sometimes multiple, sensory systems including magnetite-based, photo-chemical mechanisms, lateral lines and ampullae of Lorenzini^3,4^. Magneto-sensitive animals respond to small changes in the inclination, intensity and/or direction of a magnetic field^4^. They employ either a magnetic compass and/or magnetic map enabling homing and/or migration over short and long distances^5^. Electro-sensitive species are able to detect weak electric fields used to detect prey and predators, to communicate, find mates and/or locally orientate^6^. Electro-sensitive species are also able to respond to magnetic fields using electro-sensory apparatus and some species may have both electro and magneto-sensory apparatus^7^. While we are still trying to understand the mechanisms involved in EM-sensing^4,7^, the functional roles are clearly of fundamental ecological importance.

Interference with animal’s sensory abilities is associated with anthropogenic activity (e.g. increased acoustic noise affecting fish^8–10^, changing songs of birds^11^ and frogs^12^ and light pollution affecting the ecology of birds, turtles and fish^13^). Anthropogenic EMFs, represent a poorly understood, yet potentially important and increasing emission into the marine environment, which may disrupt or mask vital environmental cues to EM-sensitive species.

Ubiquitous anthropogenic sources of marine EMFs include ships, bridges and subsea cables^1,14^. With growing socioeconomic importance^15^, subsea cables are increasing worldwide in number, capacity, and extent with advances in electrical power generation, SMART grids, interconnector transmission and telecommunications^1,16^. Of particular interest are electricity cables given the global commitment to offshore wind and marine renewable energy and new technologies for offshore floating wind energy^17,18^. The transfer of electricity, either by direct current (DC) or alternating current (AC) cables, emits an EMF^19^. Modern cable sheathing retains the electric field but the DC or AC magnetic field is emitted into the surrounding environment and from that arises a motionally induced electric field (DC or AC) either from the rotational nature of an AC magnetic field^20^ and/or from water/animal movement through the AC/DC magnetic field^21^. However, the strongest AC electrical fields are caused by eddy currents as the result of the AC magnetic field. Short distance energy transmissions typically use AC cables but longer distances and greater capacity are more suited to DC cables, which are expected to be preferred in future energy installations further offshore^22^. Subsea cables can be free in the water column but are more commonly laid with protection on, or buried in the seabed^23^, however this does not shield the emitted EMF, which is often used to locate buried cables^24^.

Here we characterise the EMF environment associated with two subsea, buried, high voltage direct current (HVDC) cables using custom-built instrumentation to simultaneously measure the magnetic and electric field. The *in situ* measurements were taken at the Cross Sound Cable (CSC), which runs between Connecticut and Long Island and at the Neptune Cable that connects New Jersey and Long Island, USA. These measurements are used to develop models of the EMF, which can assist in understanding the EMF from future, higher capacity cables. We then assessed the behavioural response of two bottom-dwelling marine animals, to the EMF environment created by a HVDC cable.

Since bottom-dwelling EM-sensitive organisms are most likely to encounter the EMF of subsea buried cables^25^, we focused on two benthic species of ecological and commercial importance. The Little skate, *Leucoraja erinacea*, is a good model organism for the electro-sensitive elasmobranchs, with a well-understood sensory system used when foraging^26–30^ and that exhibits short distance onshore/offshore seasonal migrations^31^. The American lobster, *Homarus americanus*, is a commercially valuable species^32^ and is thought, due to its home range, coastal movement and onshore/offshore seasonal migration^33,34^, to potentially have magneto-sensory abilities similar to the Caribbean Spiny lobster, *Panulirus argus*^35,36^.

To assess if each species change their movement behaviour in response to EMF, an *in situ* enclosure experiment was designed allowing high frequency, three dimensional, fine-scale tracking of an individual’s position when exposed to the EMF and compared with exposure to a control (no EMF). It is possible that animals may explore the EMF, be attracted to or avoid it, either by swimming over the EMF and/or showing restricted movement. These ecologically relevant behaviours can be deduced from assessments of the distance travelled, speed of movement, frequency of changes in direction (turns) and the height from the seabed. Additional information can be gained from assessing these behaviours over the gradient of EMF (zones).

Assessing behavioural responses in EM-sensitive species is a first step to determining if these animals respond to the anthropogenic EMF from a HVDC cable *in situ*. Determining if there is an effect of the EMF, is an important step in the process of considering whether it could become an environmental impact^37^. The ecological insight from these behavioural studies, together with the direct measurement and modelling of the EMF from these HVDC cables, will be important for considering future deployment of subsea cables. Furthermore it will provide direction towards the research needs regarding anthropogenic EMFs and EM-sensitive animals in the marine environment.

## Results

### Animal study

#### Enclosure environment

In the enclosures (lwh: 5.0 × 3.5 × 2.5 m), the environmental parameters were similar for both skate (Temperature: $\bar{{\rm{x}}}$ = 18.8 °C (sd = 0.72), Salinity: $\bar{{\rm{x}}}$ = 29.3 psu (sd = 0.22), DO: $\bar{{\rm{x}}}$ =  8.7 mg l^−1^ (sd = 0.75)) and lobster (Temperature: $\bar{{\rm{x}}}$ = 24.0 °C (sd = 0.85), Salinity: $\bar{{\rm{x}}}$ = 29.2 psu (sd = 0.21), DO: $\bar{{\rm{x}}}$ =  6.7 mg l^−1^ (sd = 0.71)) studies. Temperature increased with the lobster release group (collinear, variance inflation factor >3) but this was not found in the skate study. Mean current speed was 0.4 m s^−1^ (sd = 0.3). The only known difference between the control and treatment enclosures was the EMF emitted by the electrical power transmission cable.

In total, during the skate study, the cable was powered (i.e. >0 MW) for 62.4% of the time with the mean power level during the exposure period being 118 MW (sd = 94.32). The electrical power varied between 0 and a maximum of 330 MW; 0 (37.5% of the time), 100 (28.6%) and 330 MW (15.2%), corresponding to electrical currents of 16, 345, and 1175 Amps. The maximal magnetic fields on the seabed in the treatment enclosure at these power levels were 51.6, 55.3 and 65.3 μT, respectively, which is a maximal positive deviation of 0.3, 4.0 and 14 µT from the Earth’s magnetic field (51.3 µT).

The power in the cable during the lobster study was constant at 330 MW (1175 A, max 65.3 μT).

#### Spatial distribution of skates and lobsters

Animals used the full length of the enclosure and spent time in each of the spatially defined sections (i.e. 40 spatial bins, Supplementary S1). **Skates:** The spatial distribution of time spent in each section of the enclosures (bins 1–40) was similar in that the skates spent most of their time at the ends of each enclosure (n = 8, D = 0.250, *p* = 0.139, Supplementary S2). However, reducing the dataset to remove the ‘end effect’ shows that skates spent significantly less time in the central sections (bins 7–34) of the treatment enclosure compared to the control (n = 8, D = 0.393, *p* = 0.019. **Lobsters:** The time spent by lobsters in sections throughout the enclosure (bins 1–40) differed between the enclosures (n = 13, D = 0.325, *p* = 0.022, Supplementary S2) and treatment lobsters spent more time in the central space (bins 7–34) than they did at the control enclosure (n = 13, D = 0.464, *p* = 0.003).

#### Behaviour in response to EMF

The statistical models fitted to behavioural data are summarised in Table 1 together with the statistical significance of the factors retained in the best fit minimal model. The model output was back transformed where necessary and used to plot the relationship with 95% confidence intervals and that relationship is described for each species.

**Table 1:** Statistical model summary.

Parameter	Model type	Fixed factors:				Random intercept = Group
		Enclosure	Sequence	Enc.*Seq.		
**Skate behaviour models**						
Total distance travelled^a^	gls	**<0**.**001**	0.343	**0**.**013**	N.S.	
Mean speed of movement^a^	gls	0.0830	0.129	**0**.**051**^e^	N.S.	
Proportion of large turns	glm^b^	**<0**.**001**	**0**.**004**	N.S.	N.S.	
Height from seabed^a^	gls	**<0**.**001**	N.S.	N.S	N.S.	
**Lobster behaviour models**						
Total distance travelled	glm	0.077	N.S.	N.S.	0.005	
Mean speed of movement^a^	glm	0.646	N.S.	N.S.	0.005	
Proportion of large turns	glmmPQL^c^	0.122	0.957	**0**.**004**	Yes^d^	
Height from seabed^a^	gls	**<0**.**001**	N.S.	N.S.	N.S.	

A summary of the statistical models fitted to describe the skate and lobster behaviour and the significant parameters in the best fit minimal models (*p* values are reported or N.S. for not significant where factors were dropped from the model). Model types include: ‘gls’ generalised least squares, ‘glm’ generalised linear models, ‘glmmPQL’ generalised linear mixed effect model using Penalized Quasi-likelihood. Non-significance of the fixed factors or random intercept is indicated by ‘N.S’.

^a^These behavioural parameters were log transformed to meet the assumptions of model fitting.

^b^Glm with quasi-binomial family.

^c^GlmmPQL with binomial distribution.

^d^Random structure was retained based on comparison of glmmPQL and gls, no *p*-value generated.

^e^Significance was borderline but retaining the parameter improved the fit of the model.

#### Skate behaviour

**Figure 1:**
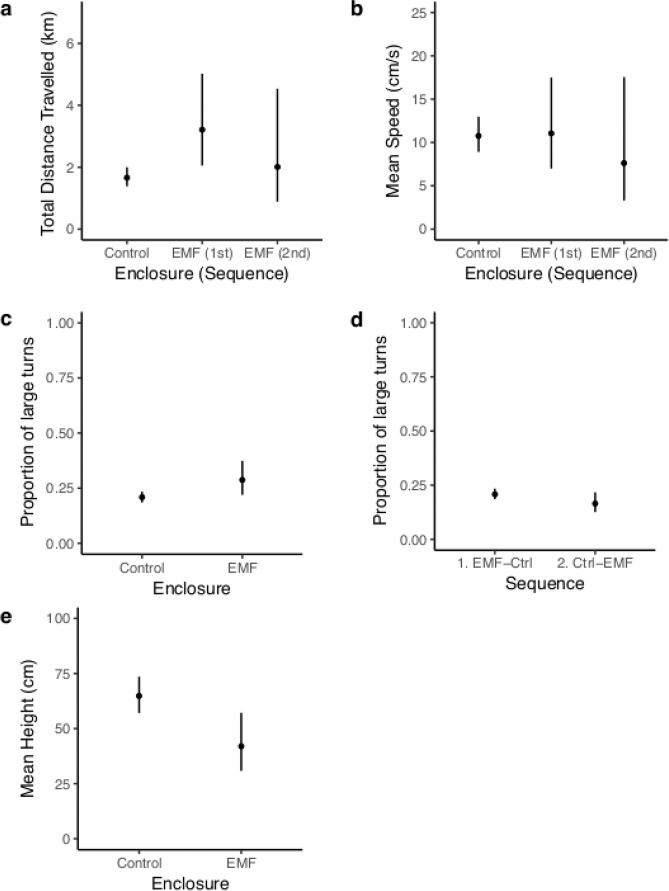
Skate behaviour in response to EMF. (**a**) The modelled estimates of the mean total distance travelled by skates in each enclosure as influenced by the sequence of exposure to the enclosures. (**b**) The modelled estimates of the mean speed of movement by skates at each enclosure as influenced by the sequence of exposure to the enclosures. (**c**) The modelled estimates of the mean proportion of large (170–180°) turns by skates at each enclosure; and for each sequence (**d**). (**e**) The modelled estimates of mean height of the skates from the seabed at each enclosure. The estimates were back-transformed where appropriate and the 95% confidence intervals are shown. The maximum EMF at the base of the treatment enclosure was 65.3 µT and the control enclosure was 51.3 µT.

There were significant differences in the total distance travelled by skates, their speed of movement, proportion of large turns and their height from seabed when compared between the behaviour in the control and treatment enclosure, with some influence of the sequence of exposure to EMF (Table 1). **Distance:** The estimated mean total distance travelled by control skates was 1.66 km whereas they travelled 3.21 km in the treatment enclosure (Fig. 1a). The sequence of exposure to the enclosures influenced the distance travelled. When treatment skates were first in the sequence, the distance travelled ranged from 2.05–5.02 km (based on the 95% CI from the model; Fig. 1a; Treatment, 1st); representing an increase of up to 93% compared to control skates. The increase in distance travelled was less pronounced in skates that had been exposed to the control enclosure prior to the treatment enclosure; they travelled 0.22 km further, which is an increase of 21% compared to control skates. In this case, the 95% CI from the model were broader with the distance travelled ranging from 0.89–4.54 km (Fig. 1a). **Speed:** The estimated mean speed of movement by control skates was 10.75 cm s^−1^ (95% CI: 8.90–12.98 cm s^−1^). The sequence of exposure to the enclosures influenced the mean speed of movement. When the treatment enclosure was first in the sequence, skates travelled at 11.07 cm s^−1^ (95% CI: 7.00–17.50 cm s^−1^); the treatment skates moved 3% faster than the control skates (Fig. 1b). Treatment skates exposed to the control enclosure prior to the treatment moved at a mean of 7.62 cm s^−1^ (95% CI: 3.30–17.55) which is 29% slower than the control skates. Skates moved faster in the enclosure second in the sequence of exposure, regardless of enclosure. However, the increase was a magnitude larger when the second enclosure was the treatment compared to when it was the control. **Proportion of large turns:** The estimated mean proportion (bound between 0 and 1) of 170–180° turns at the control enclosure was 0.21 (95% CI; 0.18–0.23) while at the treatment enclosure it was 0.29 (95% CI; 0.22–0.37) (Fig. 1c). The treatment skates used a 38% higher proportion of large turns than those at the control enclosure. Independent from the enclosure, the two sequence groups also showed a significant difference in the proportion of large turns (Fig. 1d). For the skates from Sequence 1, (i.e. treatment then control) the proportion of large turns was 0.21 (95% CI; 0.18–0.23) whereas for skates from Sequence 2 (i.e. control then treatment), the proportion of large turns was 0.17 (95% CI; 0.13–0.22). Therefore skates from Sequence 2 showed 20% lower proportion of large turns. **Height:** The estimated mean height from the seabed of control skates was 64.68 cm (95% CI; 57.05–73.55) while treatment skates were on average 41.96 cm (95% CI; 30.81–57.16) from the seabed (Fig. 1e). The treatment skates were 35% closer to the seabed.

#### Lobster behaviour

**Figure 2:**
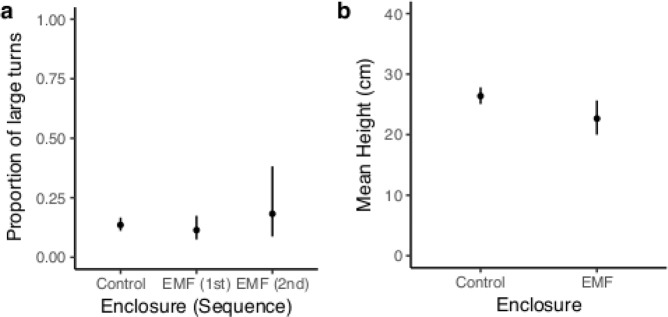
Lobster behaviour in in response to EMF. (**a**) The modelled estimates of the mean proportion of large (170–180°) turns by lobsters at each enclosure, as influenced by the sequence of exposure to the enclosures. (**b**) The modelled estimates of mean height of the lobsters from the seabed at each enclosure. The estimates were back-transformed and the 95% confidence intervals are shown. The maximum EMF at the base of the treatment enclosure was 65.3 µT and the control was 51.3 µT.

Significant differences were found in the proportion of large turns made by lobsters and their height from seabed when compared between the control and treatment enclosure with some influence of the sequence of exposure (Table 1). **Distance:** The mean total distance travelled by lobsters per day was similar at each enclosure with only 7% difference (Control: 4.05 km (95% CI; 3.75–4.35 km), Treatment: 3.76 km (95% CI; 3.13–4.38 km). **Speed:** The estimated mean speed of lobster movement at each enclosure was similar with only 3% difference (Control: 10.14 cm s^−1^ (95% CI; 9.06–11.34), Treatment 10.41 cm s^−1^ (95% CI; 8.28–13.10)). **Proportion of large turns:** The estimated mean proportion of large turns by control lobsters was 0.14 (95% CI; 0.11–0.17) while for treatment lobsters (1^st^ in the sequence) it was 0.11 (95% CI; 0.07–0.17). The treatment lobsters showed a 16% lower proportion of large turns than control lobsters (Fig. 2a). For the treatment lobsters (2^nd^ in the sequence), the proportion of large turns was 0.18 (95% CI; 0.08–0.23); a 34% higher proportion of large turns (Fig. 2a). Lobsters used an increased proportion of large turns at the enclosure second in the sequence of exposure, regardless of enclosure. However, this trend was stronger when the second enclosure was the treatment compared to when it was the control. **Height:** The estimated mean height of control lobsters from the seabed was 26.40 cm (95% CI; 25.06–27.81) while treatment lobsters were 22.65 cm (95% CI; 20.00–25.66) from the seabed (Fig. 2b). Treatment lobsters were closer to the seabed by 14%.

#### Comparing animal behaviour between enclosures and EMF zones

The cable crossed the enclosure, off-centre and at an 86° angle; approximately perpendicular to the long side. This presented a gradient of EMF within the treatment enclosure allowing two zones of high (>52.6 µT) and low (<49.7 µT) EMF to be spatially defined (Supplementary S3) and comparable spatial zones were defined at the control enclosure (both 51.3 µT). Statistically significant behavioural parameters (Table 1) were analysed to determine if animal behaviours were associated with high (>52.6 µT) or low (<49.7 µT) EMF (different zones; calculations were proportional to their aerial extent; Supplementary S3) at the treatment enclosure, by calculating the difference between zone 1 and zone 2, and comparing the difference between the two enclosures. Low EMF is caused by the magnetic field induced by the cable electrical current cancelling, in part, the Earth’s magnetic field, thus, lowering the total field.

**Figure 3:**
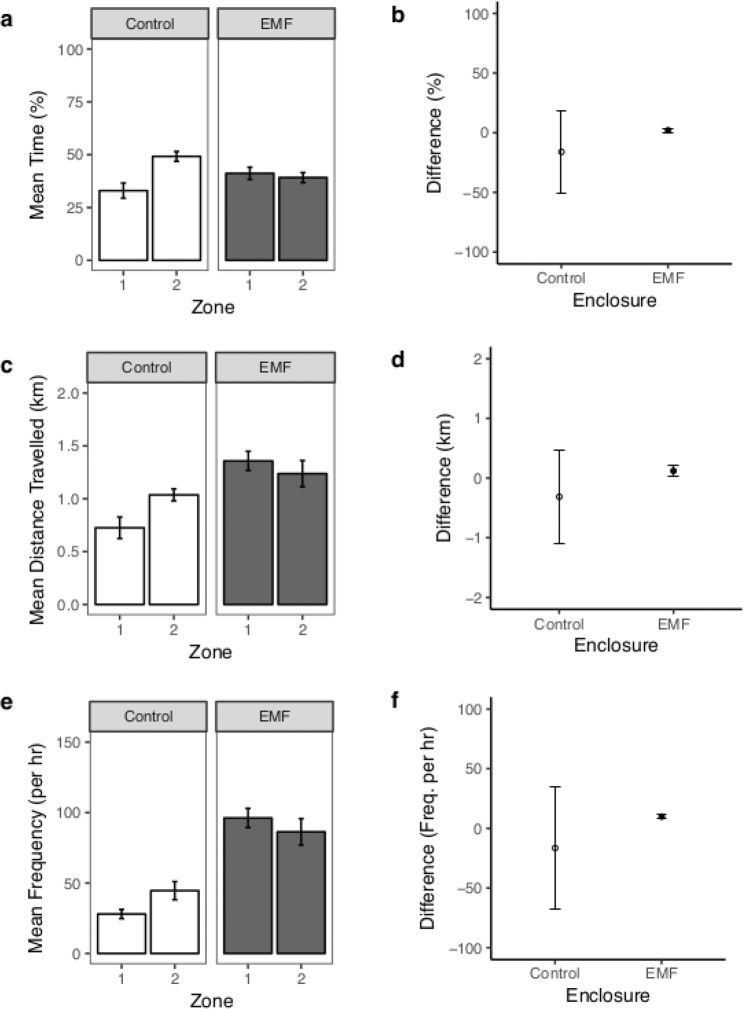
Skate behaviour in each zone of the enclosures. (**a**) The group mean (±SE) of the mean proportion of time skates spent in each zone (Zone 1 > 52.6 µT, Zone 2 <49.7 µT) at each enclosure, with (**b**) the arithmetic mean difference (±95% CI) in time spent in each zone (i.e. Zone 1- Zone 2) at each enclosure. (**c**) The group mean (±SE) of the total distance travelled per day by skates in each zone at each enclosure with (**d**) the arithmetic mean difference (±95% CI) in distance travelled in each zone at each enclosure. (**e**) The group mean (±SE) of the frequency of large turns per hour by skates in each zone at each enclosure with (**f**) the arithmetic mean difference (±95% CI) in the frequency of large turns per hour in each zone at each enclosure. Note, the comparison is firstly the difference between zone 1 and 2 in each enclosure and then between the enclosures.

**Skate:** Comparing between treatment and control enclosures indicated that the skates spent a greater amount of time in zone 1 (high EMF (>52.6 µT) at the treatment enclosure (n = 8, t = −2.366, df = 13.9, p = 0.033, Fig. 3a,b). Within the zone of high EMF the skates also travelled further (n = 8, t = −2.662, df = 13.6, p = 0.019; Fig. 3c,d) and exhibited a higher frequency of large turns (n = 8, t = −2.284, df = 14, p = 0.039; Fig. 3e,f). There was no significant difference in mean skate speed of movement (n = 8, t = 1.476, df = 9.8, p = 0.171) or the height from seabed (n = 8, t = 0.355, df = 9.0, p = 0.731).

**Lobster:** The proportion of large turns (n = 13, t = −0.479, df = 23.1, p = 0.636) and the height from seabed did not differ between zones within the enclosures (n = 13, t = −1.410, df = 21.7, p = 0.173).

#### Summary of animal behaviour in response to EMF

The results of this study clearly demonstrate that there were multiple statistically significant differences in the behavioural parameters assessed, when exposed to the EMF of the CSC, in both skates and lobsters. The basic comparison made is between the behaviour of animals in the control enclosure to the behaviour of animals in the treatment enclosure where they were exposed to an EMF from the cable. The analyses completed also account for the grouping structure and sequence of exposure to the two enclosures.

The assessment of spatial distribution patterns (Supplementary S2) showed that skates and lobsters both used the full extent of the enclosures, however, they spent significantly different periods of time in the central area of the two enclosures. The strongest behavioural response to EMF was found in the electrosensitive species, the Little Skate, where they were observed to differ significantly in the distance travelled per day, speed of movement, their height from seabed, and proportion of large turns. Furthermore, in the skates, the different pattern of spatial distribution, distance travelled and proportion of larger turns was associated with the zone of high EMF (>52.6 µT). These analyses are summarised for each skate behavioural parameter in Table 2. The lobsters, a putative magneto-sensitive species, also demonstrated statistically significant responses to the EMF, in the proportion of large turns and height from seabed. There was however no indication that either of these parameters were associated with zones of high or low EMF but was an overall response. The lobster analyses are summarised for each behavioural parameter in Table 3.

**Table 2:** Summary of skate behaviour.

Behavioural Parameter	Statistically significant	Summary
Spatial Distribution	**Yes**	Skates used the full available space in both enclosures, and they spent most of their time at the ends of the enclosure. However, skates spent more time in the central space of the control enclosure compared to the treatment enclosure.The skates spent more time in zone 2 at the control enclosure, whereas there was no difference in their distribution across zones 1 and 2 at the treatment enclosure. Comparing the difference in the use of zones between enclosures indicated that the skates spent a greater amount of time in zone 1, the zone of high EMF (>52.6 µT), at the treatment enclosure, compared to zone 1 in the control enclosure.
Total Distance Travelled (per day)	**Yes**	The skates travelled further at the treatment enclosure. This effect was more pronounced when they were exposed to the treatment enclosure first (93%) than when they were exposed to the treatment enclosure second in the sequence (21%). The distances travelled in each zone differed significantly when compared between enclosures; the skates travelled further in the zone of high EMF (>52.6 µT) at the treatment enclosure.
Speed of Movement	**Yes**	Skates moved faster within the enclosure second in the sequence of exposure, regardless of which enclosure that was. However, the difference was larger when the second enclosure was the treatment where the increase was 29% compared to when the second enclosure was the control, where there was a slight increase of 3%. There was however no indication that the change in mean speed was associated with zones of high (>52.6 µT) or low (<49.7 µT) EMF.
Proportion of Large Turns	**Yes**	At the treatment enclosure, the skates exhibited a significantly higher proportion of large turns (38%) compared to the control enclosure. Skates exhibited a higher proportion of large turns in Zone 2 at the control enclosure, but the reverse was true at the treatment enclosure indicating that the proportion of large turns was associated with the zone of high EMF (>52.5 µT). Independent of the enclosure, skates from Sequence 1 exhibited a higher proportion of large turns (20%) than those from Sequence 2.
Height from Seabed	**Yes**	Skates were on average closer to the seabed (35%) at the treatment enclosure compared to the control enclosure. There was however, no indication that being closer to the seabed was associated with high (>52.6 µT) or low (<49.7 µT) EMF zones.

The findings from multiple statistical analyses are drawn together to summarise the findings of the changes in behaviour that were found when comparing the behaviour of skates between the two enclosures (control and treatment).

**Table 3:** Summary of lobster behaviour.

Behavioural Parameter	Statistically significant	Summary
Spatial Distribution	**Yes**	Lobsters used the full available space in both enclosures, and they spent most of their time at the ends of the enclosure. However, they spent more time in the central space of the treatment enclosure and had a different pattern of distribution compared with the control enclosure. This difference in distribution pattern was consistent regardless of the sequence of release into the enclosures. There was no indication that this pattern was related to zones of high (>52.6 µT) or low (<49.7 µT) EMF.
Total Distance Travelled (per day)	No	There were no significant differences in the total distance travelled by lobsters when compared between the control and treatment enclosures.
Speed of Movement	No	There were no significant differences in the speed of movement by lobsters when compared between the control and treatment enclosures.
Proportion of Large Turns	**Yes**	The lobsters exhibited a higher proportion of large turns in their direction of travel at the enclosure that they went to second in the sequence, and this observation was most pronounced at the treatment enclosure when it was second in the sequence. There was however, no indication that the increased proportion of large turns was associated with high (>52.6 µT) or low (<49.7 µT) EMF.
Height from Seabed	**Yes**	The lobsters at the treatment enclosure were found to be significantly, but marginally closer to the seabed however there was no indication that this was associated with zones of high or low EMF.

The findings from multiple statistical analyses are drawn together to summarise the findings of the changes in behaviour that were found when comparing the behaviour of lobsters between the two enclosures (control and treatment).

#### Enclosure EMF

**Figure 4:**
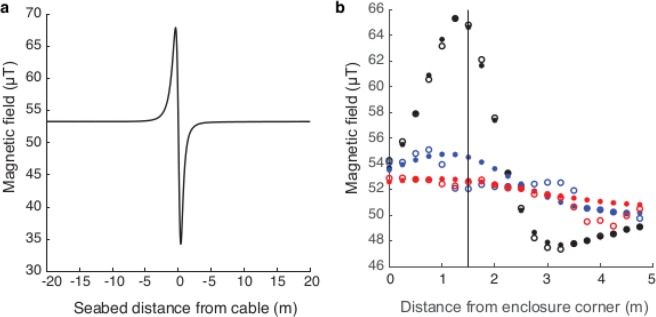
The measured and modelled magnetic field at the treatment enclosure. (**a**) The measured magnetic field of the CSC transect which was targeted for the treatment enclosure to be positioned. The maximum deviation of the Earth’s magnetic field was 18.7 μT. The electric current in the cable was 345 A. (**b**) The measured (open circles) and modelled (filled circles) magnetic field inside the enclosure. The optimization was done on the magnetic field measured at the seabed (black) and then modelled for the mid (blue) and top (red) of the enclosure. The long side of the enclosure starts a 0 m and ends at 5 m.

The measured magnetic field is the result of a superposition of the Earth’s magnetic field and the cable-generated field, which introduces an asymmetry between the two sides of the cable (Fig. 4a). The peak magnetic field at the seabed (i.e. enclosure base) was 65.3 μT (max); a clear deviation from the Earth’s magnetic field (51.3 µT). The treatment enclosure was positioned at 94° relative to the cable direction. The peak fields at mid and top height of the enclosure were weaker (55 and 53.5 µT respectively). There was good agreement of measured and modelled (Supplementary S4) magnetic fields of the base, mid and top of the enclosure (Fig. 4b). The model (Supplementary S4) indicates that the two bundled cables were placed at 120° relative to the vertical direction, with 0.1 m separation. The burial depth was estimated to be 1.3 m (4.4 feet). The model reveals that the cable was positioned 0.25 m from the maximum magnetic field level towards the centre of the enclosure (Fig. 4b, vertical line). For comparison, animals at the control enclosure were exposed to ambient magnetic fields (51.3 µT).

### Electromagnetic Fields of HVDC cables

#### Variation in EMFs with electrical current

Measurements of the EMF were taken near to the seabed by sledging the sensors over the cable. For both the Cross Sound Cable (CSC) and Neptune Cable (NC) the DC magnetic field and an unexpected AC (magnetic and electric) field were detected. The variation in each field with the operating electrical current in the cable is described.

The CSC (highest nominal current 1175 A) was measured at a maximum of 345 A where the maximum deviation of the total magnetic field (DC), relative to the Earth’s magnetic field, was 18.7 µT (negative). The average deviation of the total magnetic field (DC) was considerably higher at 345 A than when the current was 16 A (Table 4). The average positive and negative deviation of the magnetic DC field at 345 A was 3.79 and 2.83 μT and at 16 A was 0.40 and 0.28 μT, respectively (Table 4). These observations show that electric current in the cable generates deviations comparable to the strength of the Earth’s magnetic field.

**Table 4:** The measured electromagnetic field of the Cross Sound Cable (CSC) and Neptune Cable (NC).

	Positive deviation of total magnetic field, DC (µT)	Negative deviation of total magnetic field, DC (µT)	Amplitude of total magnetic field, AC (µT)	Amplitude of total electric field, AC (V/m)
**CSC**, **Powered**, **345** **A (n** = **11)**				
Average	3.79	2.83	0.15	7.22e-04
St. dev.	±3.66	±5.27	±0.12	±1.1e-04
Max	14.30	18.70	0.51	9.70e-04
Median	2.33	1.20	0.11	6.70e-04
**CSC**, **Not powered**, **16** **A (n** = **12)**				
Average	0.40	0.28	0.14	7.35e-04
St. dev.	±0.15	±0.10	±0.033	±7.9e-05
Max	0.64	0.43	0.18	8.4e-04
Median	0.35	0.30	0.13	7.3e-04
**CSC**, **Shut-down**, **0** **A (n** = **9)**				
Average	0.18	0.12	No	Not detectable
St. dev.	±0.13	±0.2	n/a	n/a
Max	0.46	0.66	No	Not detectable
Median	0.18	0.06	No	Not detectable
**NC**, **Powered**, **1320** **A (n** = **33)**				
Average	6.77	2.3	0.04	4.2e-04
St. dev.	±3.7	±2.1	±0.02	±8.7e-05
Median	2.75	1.4	0.004	4.0e-04
Max	20.7	8.3	0.09	6.5e-04
**NC**, **Powered**, **660** **A (n** = **12)**				
Average	3.0	0.9	0.023	2.4e-04
St. dev.	±0.78	±0.84	±0.005	±0.34e-04
Median	2.75	0.65	0.022	2.3e-04
Max	4.7	3.3	0.037	3.1e-04

The CSC (330 MW, 300 kV) HVDC cable was measured with a current of 345, 16 and 0 A and the NC (660 MW, 500 kV) HVDC was measured with a current of 1320 and 660 A.

The average AC fields at 345 A were 0.15 μT (AC magnetic, Table 4) and 0.72 mV/m (AC electric, Table 4). These values were comparable to levels obtained at 16 A, which were 0.14 μT and 0.74 mV/m (Table 4).

During the CSC shut down (0 A), the average DC field was twice as weak as when the electric current was 16 A. The DC field was discernible, however, there was no sign of the AC field.

When the NC operated at full power, corresponding to 1320 A, the maximal deviation was 21 μT (positive). At 1320 A, the average positive deviation of the magnetic DC field relative to Earth’s magnetic field was 6.77 μT and the average negative deviation of the magnetic DC field was 2.3 μT (Table 4). At 660 A, the average positive magnetic deviation of the DC field relative to Earth’s magnetic field was 3.0 μT and the average negative deviation of the magnetic DC field was 0.9 μT (Table 4).

At 1320 A, the average magnetic AC field was 0.04 μT and the average electric AC field was 0.42 mV/m (Table 4). When the current was 660 A, the corresponding AC fields were 0.023 μT and 0.24 mV/m (Table 4).

#### Spatial extent, symmetry of signals and harmonics of the EMF

The DC magnetic fields typically extended 5–10 m on either side of the cable. As reported in Table 4, the magnitude of the positive and negative deviation of the total magnetic field (DC) differed and resulted in an asymmetrical field (Fig. 4 & Supplementary S5). This pattern is due to the rotation of the cable pair in the cable relative to the vertical axis.

**Figure 5:**
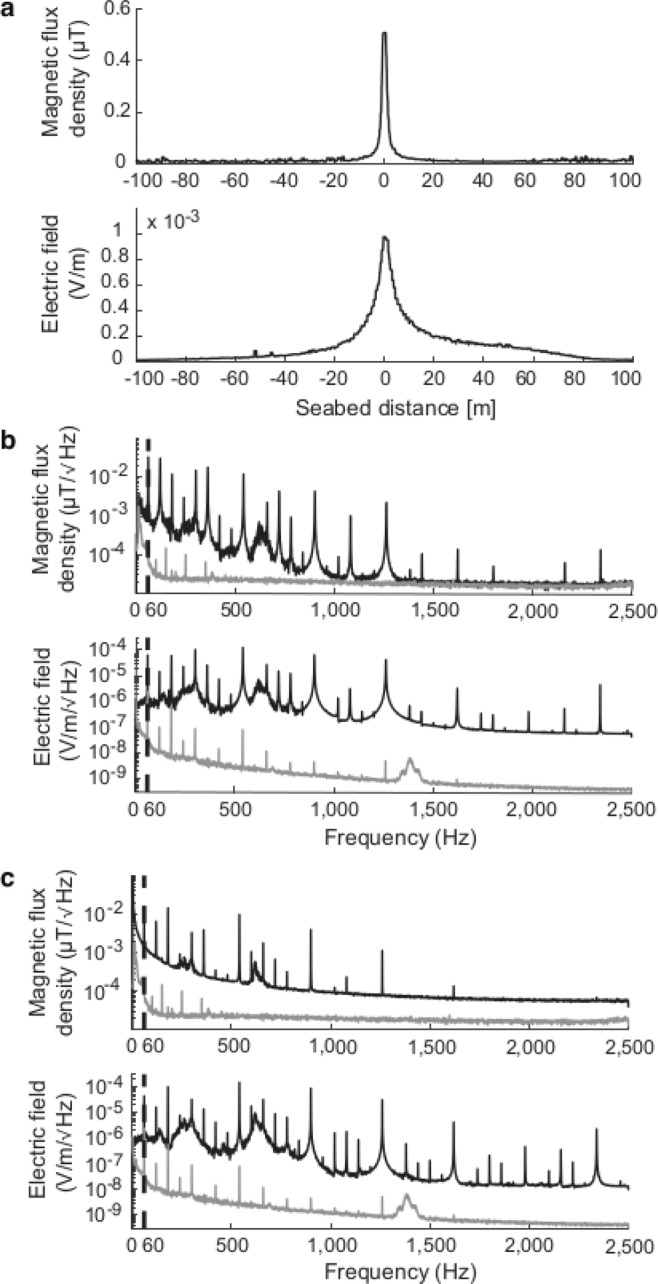
The spatial extent and harmonics of the AC fields, exemplified by the CSC. (**a**) The spatial extent of the measured AC fields; the total magnetic AC field (upper) and the total electric AC-field (lower). (**b**) The estimated spectra from the Power Spectral Densities (PSD for transect 7, black lines) during operation at 345 A; the magnetic field (upper) and the electric field (lower). The grey lines show measured background levels. (**c**) The estimated spectra from the PSD of transect 5 when the CSC was not transferring power but had an electric current of 16 A. In (**b**,**c**), the upper panel shows the magnetic AC field (black) and the lower panel the electric AC field (black). Both have the main frequency of 60 Hz identified by a dotted line and the grey lines in (**b**,**c**) show the background levels obtained at the reference site (358 m from the cable).

Generally, the widths of the electric fields were larger than the corresponding magnetic fields. For example, in Fig. 5A the reduction of measured fields to 10% of the maximum field strength, was observed at approximately 8 m from the maximum for the magnetic field and 48 m from the maximum for the electric field. The AC fields were approximately symmetrical in shape. The frequency of the measured AC fields ranged from >1 Hz (i.e. extremely low frequency) to <2500 Hz, which is also the extent of the SEMLA’s sensitivity range for electric fields. In the CSC the dominating frequency for the magnetic AC field was 60 Hz, followed by 180, 540 and 120 Hz harmonics and for the electric AC field 540 Hz followed by 180, 900 and 60 Hz harmonics (Fig. 5b). The AC fields were detectable even when the cable was not transmitting power but had a maintenance current of 16 A (Fig. 5c); considerably higher than the background levels (grey Fig. 5b,c). In the NC the dominating frequency for both the magnetic and electric field was 720 Hz, followed by 120, 180 and 360 Hz harmonics.

#### Electromagnetic field modelling

The previously described measured fields were not only affected by the magnitude of the transmitted electric current, but also by the morphological and structural properties of the cable, and the burial depth. Two models were successfully developed to describe the DC magnetic field. To accurately predict the fields at the seabed and in the water column, a model was implemented based on COMSOL software. This model made detailed estimates of the fields, based on cable geometry and material properties, in order to compare the modelled results with measured fields. The model was, however, computationally expensive and could not be used in iterative schemes. Therefore a fast model was developed based on simplified assumptions of the cable such as infinite length and no magnetic materials. The fast model was used in an optimization mode to predict the geometry and burial depth of the cable using the measured levels at the seabed and known electric current. The same model was specifically used in forward mode to estimate the EMF in the enclosure volume based on optimized parameters (Fig. 4b). Both models were accurately parameterized based on the need to describe the DC magnetic field of HVDC cables (Supplementary S4).

Using the fast model, the burial depth of the CSC (345 A) and the NC (1320 A) was estimated from the transect crossings at constant power. The estimated minimum burial depth achieved in the CSC transects was 0.58 m and the maximum 1.74 m (x = 1.41, sd = 0.37, n = 9). The estimated minimum burial depth of the NC transects was 1.16 m and the maximum 2.62 m (x = 2.09, sd = 0.35, n = 31).

## Discussion

Anthropogenic emissions of electromagnetic fields (EMFs) from subsea electricity transmission cables are quantifiable and measured at a magnitude similar to the background geomagnetic field. These EMFs are detectable by electro- and magneto-sensitive species in the marine environment such as the ecologically relevant and commercially important benthic animals assessed here. We found an ecologically significant behavioural response to the EMF of the Cross Sound Cable in the electro-sensitive Little skate, *Leucoraja erinacea* and the presumed magneto-sensitive American lobster, *Homarus americanus*. Furthermore, through fine-scale quantification of the EMF of two HVDC cables, we characterised the DC magnetic field and subsequently developed accurate models of the DC field within the benthic environment. Unexpectedly, a strong AC magnetic and electric field was also measured from both HVDC cables. Such an AC field is not predicted by modern DC models. The quantified animal responses, measured fields and present modelling capabilities, convincingly demonstrate the importance of quantifying the EMF environment appropriately within which EM-sensitive species live. Here, we have demonstrated that both skates and lobsters are able to move through the EMF emitted from a HVDC cable; however the changes in behavioural movements may infer important ecological consequences for electro- and magneto-sensitive species.

The most striking response was that skates travelled much further when exposed to the EMF. This response was particularly pronounced in skates exposed to the treatment enclosure first, where they travelled almost twice as far (93% increase, Fig. 1a) but only moved marginally faster (3%, Fig. 1b). When exposed to the treatment enclosure second in the sequence the skates still moved further (21%, Fig. 1a) but at a much slower speed (30%, Fig. 1b). Therefore the treatment skates were moving slower but were active for longer periods of time. Naturally, skates are often found resting in depressions in the seabed during the day^31,38^, which could explain the increased use of the central space in the control enclosure. The increased distance travelled in the treatment enclosure suggests that periods of rest were less frequent when exposed to EMF. The 3D positional data confirmed that skates were swimming since they used the full vertical space available. The overall slower speeds indicate that skates spent more time on the seabed either punting (a push-glide movement using modified pelvic fins, ‘crura’)^38^ and/or swimming more slowly in midwater^39,40^. Skates were also found closer to the seabed (35%, Fig. 1e) and used a higher proportion of large turns (38%, Fig. 1b) when exposed to the EMF, further supporting the suggestion that the skates spent more time punting. The crura can function independently, allowing sharp or gradual turns, not possible when swimming using pectoral fin undulation and may also produce less bioelectric noise^38^. These attributes are important when foraging for prey using an electrosensory system and may provide a behavioural self-regulation in addition to a neurological adaptive filter^38,41^.

Collectively, the increased distance travelled at overall slower speeds, with increased large turns while being closer to the seabed is indicative of increased exploratory and/or area restricted foraging behaviour^42^ when exposed to EMF. This interpretation is further supported by the increased association with the zone of high EMF (>52.6 µT) where the skates travelled further with more large turns (Fig. 3). Ultimately, exploration/foraging with no return (locality/food) infers an energetic loss unless sensitive animals are able to distinguish between natural and anthropogenic EMFs and learn from experience^43,44^. Although some studies have noted increased elasmobranch swimming activity in response to magnetic fields^7,45^, these have been studies of conditioned behaviour to help determine detection abilities rather than natural behavioural responses to anthropogenic EMFs, so are not directly comparable. An increase in distance travelled and suggestion of area restricted movement^46,47^ was previously observed in *in situ* enclosure studies of *R*. *clavata* in response to an AC cable, emitting EMF within the range of detectability of the skate^48^, although there was no measure of the total distance travelled.

Comparatively, the lobsters exhibited a more subtle behavioural response to EMF in that they were found more frequently in the central space of the treatment enclosure, were closer to the seabed (14%, Fig. 2) and used more large turns (second exposures, Fig. 2). During this study, lobsters were observed to climb the sides of the enclosures, which can be considered natural behaviour in the enclosure setting since they are known to climb on top of rocks and also into net traps^49,50^. Lobsters are also able to ‘swim’ via cardioid escape responses^51^ and are reported to create depressions in sand and burrow in mud^52^. Therefore, lobsters exposed to EMF climbed the enclosures less and explored the seabed more, most likely foraging or in search of burrows.

As a seasonal migratory species^34,53^, *H*. *americanus* may possess a polarity compass similar to *P*. *argus*^35,36^ that could be used in homing and/or migration. To date there is no reported evidence of a magnetoreceptive sensory ability in *H*. *americanus* and there is limited anatomical research in crustaceans^54–56^. However support for a crustacean ability to detect magnetic stimuli arises from behavioural responses to the geomagnetic field e.g. the Red King crab, *Paralithodes camtschaticus*^57^, amphipods^58,59^, and an isopod^60^. Furthermore electromagnets have been shown to affect shelter choice, roaming activity and hormone levels in the edible crab, *Cancer pagurus*^61^. To understand the importance of the effect of the EMF on the behaviour of *H*. *americanus*, knowledge of the physiological ability and ecological importance of magneto-reception in *H*. *americanus* is required.

Skates and lobsters both changed their behaviour in response to the anthropogenic EMF of the CSC. The measured electromagnetic fields of the CSC and the NC demonstrated that the electric current in the cables generated magnetic fields (DC and AC) that were of a similar magnitude to variations in the geomagnetic field (Tab le 4). Thus, the magnetic fields emitted are within the presumed range of sensitivity (nT-µT) to magneto- and electro-sensitive marine animals^62^. Owing to the physics of the AC magnetic field, an induced electric field (AC, i.e. due to the alternation of the AC magnetic field) was present and detected (Fig. 5, Table 4), also within the known sensitivity range of aquatic species (<1–100 µV cm^−1^, 1–100 Hz^6,63^). The laws of physics, inform us that a motionally induced voltage (MIV) would also arise from an animal or water body passing through the anthropogenic EMF (DC or AC), however this MIV would be below the levels of detection of our instrumentation. The benthic marine species in this study, responded to the anthropogenic EMF of the CSC, however, exactly what component of the EMF, intensity and frequency they responded to (i.e. DC, AC, MIV) remains an intriguing question for the future.

The total zone affected by cable induced magnetic fields (DC and AC) in this study, was 5–10 m on either side of the cable, inferring the potential area of influence to be 10–20 m wide. The fields also extend vertically (e.g. 2.5 m, Fig. 4b), however decrease in magnitude with distance from the cable. The measured AC electric field extended approximately 100 m and may represent a larger area of potential influence for electro-sensitive species. Furthermore, the DC magnetic field was scalable to the power in the cable indicating that future higher capacity cables may produce higher magnitude distortions of the geomagnetic field.

The origin of the AC fields detected from the CSC and the NC is not known; it could be from the cables, if not perfectly grounded, but is more likely from the AC/DC converter stations^64^. Comparing the DC and AC magnetic signals, the average amplitude of the AC field was about three times weaker than the average DC field. Even at 16 A, the AC field was low but detectable however when the cable was shut down, the AC field was not detected. There was, however, still a DC magnetic field detectable from the CSC when it was shut down, most likely due to magnetization of the cable material.

The DC magnetic fields measured, deviated from the background magnetic field in the range of 0.4–18.7 μT for the Cross Sound Cable (CSC) and 1.3–20.7 μT for the Neptune Cable. The variation was attributed, primarily to burial depths, which were estimated using the models. The targeted installation depths for these cables were 1.2–1.8 m (4–6 ft). Approximately 90% of CSC and NC transects were estimated to be within the targeted burial depth yet an EMF was still discernible at magnitudes similar to geomagnetic inclinations. It is apparent from this study that when a cable is transmitting a constant power, the EMF strength will vary along the cable route due to the variation in burial depth. These results highlight the need to measure and/or accurately model the EMF and its variability at the seabed surface and in the water column, and consider these factors in any assessments of responses from receptor species.

The COMSOL model is commercially available and was accurately built based on the expected DC field. However it proved to be slow and cannot be used in an iterative fashion like the ‘fast model’, which was based on the physics of the likely relationship between the electricity transmitted and the EMF emissions. The COMSOL model is able to be scaled up to higher capacity HVDC cables making it useful in applications of future cabling scenarios and assisting with understanding future changes in anthropogenic EMF in the marine environment. The origin of the AC field must be identified and the models updated to ensure that they reflect the reality of the EMF observed in the environment.

Using a cross-disciplinary approach we have quantified and modelled changes to the marine EMF environment associated with two subsea HVDC cables and demonstrated the influence of a cable EMF on the behaviour of commercially and ecologically important bottom-dwelling species. We presented evidence that the EMF of subsea buried cables exists at an ecologically important magnitude at the seabed and in the adjacent water column, with a larger than expected area of influence that is variable along the cable route. Most importantly, we demonstrated a striking increase in exploratory/foraging behaviour in skates in response to EMF and a more subtle exploratory response in lobsters. Future research must further define the EM environment together with sensitivity thresholds and likely encounter rate of EM sensitive species in order to establish if a behavioural effect may become a population level impact^37^.

## Materials and Methods

### Experimental design

#### Animal enclosure study

Skates were collected by the University of Rhode Island (URI) fish trawl (<30 min). Lobsters were obtained from a local commercial lobster fisher under a Scientific Collectors Permit. Ethical approval was authorised by the URI Institute of Animal Care and Use Committee (URI-IACUC) and the methods were carried out in accordance with guidelines and regulations. All specimens were housed at the URI Marine Science Research Facility, prior to the field study. Specimens were maintained on a diet of squid, in a 3 m diameter tank under artificial local light regime with an aerated, sand filtered local water supply; skates were supplied with sand.

A total of 39 skates (length $\bar{{\rm{x}}}$ = 46.5 cm, SD = 2.34, plus one at 30.6 cm due to stumped but healthy tail) and 65 lobsters (carapace $\bar{{\rm{x}}}$ = 85.3 mm, SD = 1.16) were released into the enclosures in single sex groups; 13 lobster groups (male & female) and 8 skate groups (all female). Lobster familiarisation to reduce aggression was achieved by controlled staged fights (groups of 5, 3 fights, 15 min) prior to release in conspecific familiar groups. Individually coded acoustic tags were attached to a wing of the skates with Peterson discs and lobsters wore a cable-tie harness. One group of individuals (n = 2–5) was released into each enclosure (mesocosms, lwh: 5.0 × 3.5 × 2.5 m) built from non-magnetic materials. The treatment enclosure (T) was placed on top of the buried electrical transmission cable (HVDC Cross Sound Cable, Long Island Sound, USA; 41.223563, −72.900229), and the control enclosure (C) at a similar site with no cable (41.226639, −72.898889). Surveys to determine the electromagnetic fields (EMF; see ‘Electromagnetic Field Measurements’) informed site selection. The control site was 358 m from the treatment site and had similar bathymetry (10 m) and seabed type (mud/sand). The lobster study occurred in August-September and the skate study in September–October 2016.

Four hydrophones (Hydroacoustic Technology Inc. (HTI) hydrophones, Model 590) within each enclosure (Supplementary S1) triangulated the three dimensional positions of each acoustically tagged animal (HTI tag models 795-LG/LY, 2000–2999 msec period, 0.5 msec pulse width). Each array of hydrophones was hardwired to electronics (Acoustic Tag Receiver Model 291, marine batteries and transformer) housed on a floating platform (c.a. 5 × 5 m). Environmental parameters were monitored (*In Situ* Inc. Troll 9000 Pro: temperature, oxygen, salinity; 5 minute frequency). Accurate daylight regimes were recorded (timeanddate.com). A GoPro^®^ camera was mounted on the inside wall of the enclosure, angled down to view the animals on the base of the enclosure (Supplementary S1). Video data were used to qualitatively confirm the directional movements of the animals on the base of the enclosure, thereby truthing the positional data obtained from the HTI system. A stationary beacon acoustic tag mounted centrally on the internal ceiling of each enclosure provided a control of offsets. The variation in the magnetic field at each enclosure was continuously measured by two magnetometers (Bartington MAG-03 and Applied Physics System Model 544) every 5 mins. The field across the dimensions of each enclosure was also assessed during the period of constant cable power (330 MW; see ‘Electromagnetic Field Measurements’) using the Bartington magnetometer. At the base of the treatment enclosure (seabed), the magnetic field ranged from 47.8 to 65.3 µT (along the length); a maximal positive deviation of 14 µT from the Earth’s magnetic field of 51.3 µT recorded at the control enclosure.

All specimens experienced both enclosures (18–24 h in each), in alternating sequence (i.e. C-T or T-C) therefore avoiding bias due to the order of exposure to EMF. All animals were only used once. This design provided high frequency 3D tracking data of the behaviour of individuals at the control site (no EMF) versus the treatment site (EMF from cable).

#### Electromagnetic field measurements

A custom-designed instrument, the Swedish ElectroMagnetic Low-noise Apparatus (SEMLA) was employed to measure low intensity electromagnetic fields *in situ*. The SEMLA was equipped with skis (i.e. a sled) to place it close to the seabed allowing both the maximum magnetic and electric fields to be measured and to move smoothly over the seabed. Positioning on the seabed also stabilized the platform thereby reducing motion-induced noise. The SEMLA was equipped with a low-noise three-axial fluxgate magnetometer (Bartington MAG-03, Sensitivity; 6 pT/√Hz at 1 Hz, Frequency response from DC to 3 kHz), and three axial electric sensors (Polyamp AB, sensitivity of 5 nV/√Hz at 1 Hz^65^). On a flat seabed the sensors were positioned at specific heights: the fluxgate at 0.15 m, two electric sensors (horizontal plane) at 0.52 m, one central electric sensor (vertical plane) at 1.04 m. The fluxgate and electrode signals were directly fed to line drivers (housed close to the sensors to minimize electronic interference) which amplified the electric fields to 80 dB. The outputs of the line drivers were connected via umbilical cord to surface electronics where the signals were low-pass filtered at 1 kHz to avoid aliasing before being sampled with a 24-bit Analog-to-Digital converter (DEWE-43) at 5 kHz. The AD-converter was connected to a laptop allowing real-time monitoring of the measured fields when crossing of the cable.

The SEMLA with 150 m umbilical was deployed onto the seabed and towed approximately perpendicular to the buried cable, behind a slow moving vessel. The magnetic and electric fields were measured separately in the long, cross and vertical directions of the cable. The total magnetic field was measured, which is invariant to the Earth’s magnetic field making it possible to detect the cable. The electric field does not suffer from influence of strong external fields since there was no electric DC field in the area. Throughout this study, total fields were used in the analysis.

##### Cross Sound Cable (CSC)

A SEMLA survey of the CSC in Long Island Sounds, Connecticut, USA was completed in April-May 2016; a total of 23 km of towing with 32 cable crossings (transects) to map the *in situ* fields generated by the cable. During this survey, the CSC operated in three different modes; power transmission at 345 A (13 transects), not transferring power but had a maintenance current of 16 A (10 transects), and shut down, 0 A (9 transects). The background levels in the area were determined at 358 m distance from the cable (current was 345 A).

##### Neptune Cable (NC)

A SEMLA survey of the HVDC NC in Raritan Bay, New Jersey, USA was completed in August, 2017. In total 45 transects were towed to map the *in situ* fields generated by the cable. During this survey, the NC operated in two different modes; power transmission at 1320 A (33 transects) and power transmission at 660 A (12 transects).

##### Enclosure

The surveys of the CSC were used to locate sites for the treatment and control enclosures. The treatment site had the maximum EMF reading (transect 7, maximal deviation from Earth’s magnetic field was 18.7 μT). The fluxgate was detached from the SEMLA and used in standalone mode in a diver led survey to map the magnetic field in each enclosure (Supplementary S1). Measurements (12 s) were taken at 0.25 m intervals along the length of the base of the enclosure (at the seabed), mid-height (1.25 m from seabed) and top of the enclosure (2.5 m from seabed). This procedure was repeated parallel to the two longest, vertical sides of each enclosure (Supplementary S1). During the survey the electric current was 1175 A, which corresponded to full power transmission. The result of this survey was a detailed characterisation of the field in the treatment enclosure.

#### Electromagnetic field modelling

Models of the EMF of the HVDC cables (CSC and NC) were developed using a commercially available software package, COMSOL (Supplementary S4). COMSOL is a finite element analysis software that can be used to solve the EMF problem of complex models. It is able to model the physical level details of the real-world environment, such as structural, morphological components, and material properties. A fast numerical model (herein ‘fast model’) of the two bundled cables was also developed and used for optimization of the cable configuration (Supplementary S4). The fast model was employed since it can be iteratively used for predicting the optimal parameters, whereas the COMSOL model is too slow to be used for this application. In this study, COMSOL model was used to provide a more detailed and accurate analysis of the EMF, which will also provide reference values in order to verify the quick analysis results from the fast model. The fast model was used to calculate the burial depth of the CSC and NC using the transect data.

### Data Processing and Statistical Analysis

#### Animal enclosure study

Acoustic data were processed in HTI software (Acoustic Tag v6.20.10-3, Mark Tags, v07.00.00-17) to obtain 3D positions of individual animals and analysed in R (version 3.2.4^66,67^). Statistical analyses focused only on the influence of the EMF from the cable on behaviour of the skates and lobsters. Therefore, the only parameters used in statistical models, built using the behavioural data, were the enclosure (cable, no cable), the sequence of exposure to the cable and in the mixed models, the grouping of individuals.

Comparisons of spatial distribution between enclosures were assessed by a non-parametric Kolmogorov-Smirnov two sample test for the full length and central space of the enclosures (Supplementary S6). Behavioural parameters including the total distance travelled per day, the mean speed of movement, the proportion of large turns and the height from seabed were compared between enclosures using linear mixed effects models, generalised linear mixed effect model and generalised least squares models^68^ (Supplementary S6). Where appropriate, these behavioural parameters were log transformed to meet the assumptions of model fitting (Table 1). Note that the height from seabed is actually the height from the internal base of the enclosure and should be considered a relative comparison between enclosures rather than an absolute measurement. Further comparisons of behavioural parameters were made using a Welch’s two sample t-test, between two zones of EMF in each enclosure (Treatment: zone 1: ‘high’ (52.6 to 65.4 µT) and zone 2: ‘low’ (47.8 to 49.7 µT) compared with Control: zone 1 and zone 2 (both 51.3 µT); Supplementary S6).

#### Measured electromagnetic field analysis

The total magnetic and electric AC fields were derived in three steps: (1) three components of each field were high-pass filtered at 10 Hz to reduce the effect of low-frequency influence, (2) a moving maximum filter of 1 sec length was employed to extract the envelope, and (3) fields were adjusted for background levels.

Power Spectral Densities (PSD) were calculated to estimate spectral content. The PSD were estimated for the three orthogonal field components and added to give the spectrum for the total fields. The segment length of the transform was 1 second intervals to agree with the sampling frequency. This choice of interval makes the PSD-level and the tonal amplitude approximately equal (provided that the tones do not spill over into neighbouring bins). The total length of the PSD time segment was 10 seconds, which spans the main part of the peak measurement of the field. Note that the PSD can only be used as an indicator of spectral content since the signal amplitude varied considerably during the 10-second time intervals.

## Supplementary information

Supplementary file.

## Data Availability

Permissions are being sought to allow the datasets analysed during the current study to be made available in an appropriate repository on acceptance of this manuscript.
