# Supplementary material for: Anthropogenic electromagnetic fields (EMF) influence the behaviour of bottom-dwelling marine species
Source: Sci Rep. 2020 Mar 6;10:4219. doi: 10.1038/s41598-020-60793-x (PMC7060209; doi:10.1038/s41598-020-60793-x)
Supplement: Supplementary file 1 — Supplementary file. [file 41598_2020_60793_MOESM1_ESM.pdf]

Anthropogenic electromagnetic fields (EMF) influence the behaviour of bottom-dwelling marine species

Zoë L. Hutchison[1], Andrew B. Gill[2,3], Peter Sigray[4], Haibo He[5], John W. King[1]

[1]Graduate School of Oceanography, University of Rhode Island, USA

[2]PANGALIA Environmental, Bedfordshire, England, UK

[3]Cefas, Centre for Environment, Fisheries and Aquaculture Science, Suffolk, England, UK

[4]FOI, Department of Underwater Research, Stockholm, Sweden

[5]Department of Electrical, Computer and Biomedical Engineering, University of Rhode Island, USA

Section S1: Spatial Dimensions relating to the Animal Enclosures

Spatial bins: The animal enclosures at the control and treatment sites were 5 x 3.5 x 2.5m (l, w, h). To statistically explore the temporal and spatial distribution of the animals within the enclosures, the length of the enclosure (y-axis) which was approximately perpendicular to the cable, was split into 40 spatial bins (c.a. 13cm each, Figure S1a). To assess the distribution of animals away from the potential influence of the ends of the enclosure, a subset of the data was also analysed which represented the central space of the enclosure (bins 7 to 34 of 40).

Hydrophone geometry and controls: The geometry of the hydrophones was developed in conjunction with the HTI specialist team. We adopted a two up, two down configuration as depicted in Figure S1b. The exact separation between the hydrophones in the x, y and z dimensions were measured (in cm) prior to deployment and these dimensions were used in the software for processing the three dimensional positions of the animals. A beacon tag acting as a control, was added to the ceiling of each enclosure and remained in place and on throughout the full experiment. This control tag was used to ensure that we had full detection from all hydrophones at all times of the study and was also used to determine the error in each dimension ($x < 2$ cm, $y < 1.5$ cm, $z < 4$ cm). Importantly, comparative enclosures (i.e. C and T) had < 0.5 cm error between each dimension making them highly accurate for the purposes of behavioural comparisons. In addition, a go-pro was mounted on the internal wall angled at the base of the enclosure to capture animal movements and truth acoustic data. A total of 77 directional movements from 17 individuals were captured on camera and confirmed to be accurate according to the acoustic data. It was important that these control measures were not on the base of the enclosure to prevent them becoming a point of interest to the benthic animals.

Diver surveys: As detailed in the Methods, to fully characterise the EMF in the enclosure, a diver survey completed using the fluxgate detached from the SEMLA and used in standalone mode in a diver led survey to map the magnetic field in each enclosure. Measurements (12s) were taken at 0.25 m intervals along the length of the base of the enclosure (at the seabed), mid-height (1.25 m from seabed) and top of the enclosure (2.5 m from seabed). The positions of the diver transect surveys are shown in Figure S1c.

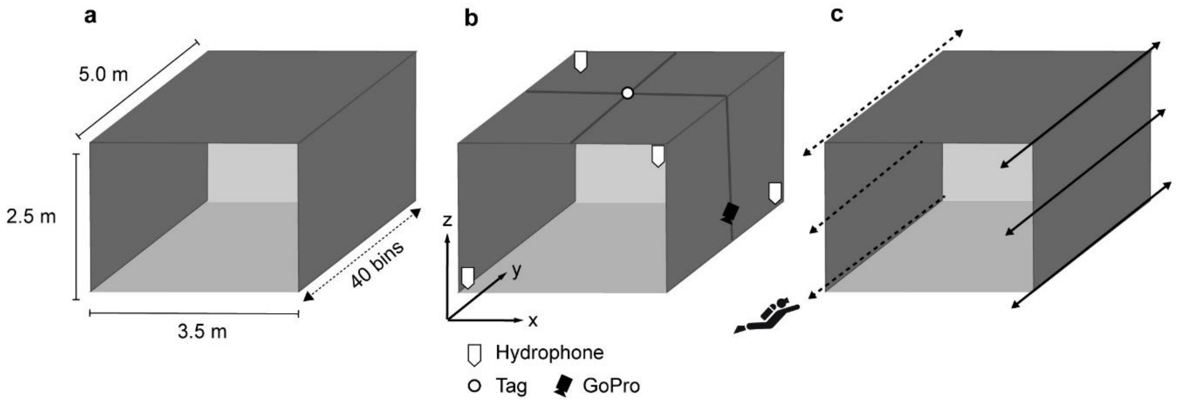

Figure S1. Spatial dimensions of the animal enclosures. (a) The animal enclosures at the control and treatment sites were 5 x 3.5 x 2.5m (l, w, h); to statistically explore the spatial distribution of the animals within the enclosures, the length of the enclosure was split into 40 spatial bins. (b) The hydrophones were mounted internally, in a two up, two down configuration with a beacon tag (control) mounted on the internal ceiling of each enclosure and a GoPro on the internal vertical wall orientated toward the base of the enclosure. (c) Diver surveys were taken along the two long vertical sides (y-axis) of each enclosure at the base (0 m from seabed), mid-height (1.25 m from seabed) and the top (2.5m from seabed). Diagrams are not to scale.

<u>Section S2: Supporting Figure for Spatial Distribution of Animals within Enclosures</u>

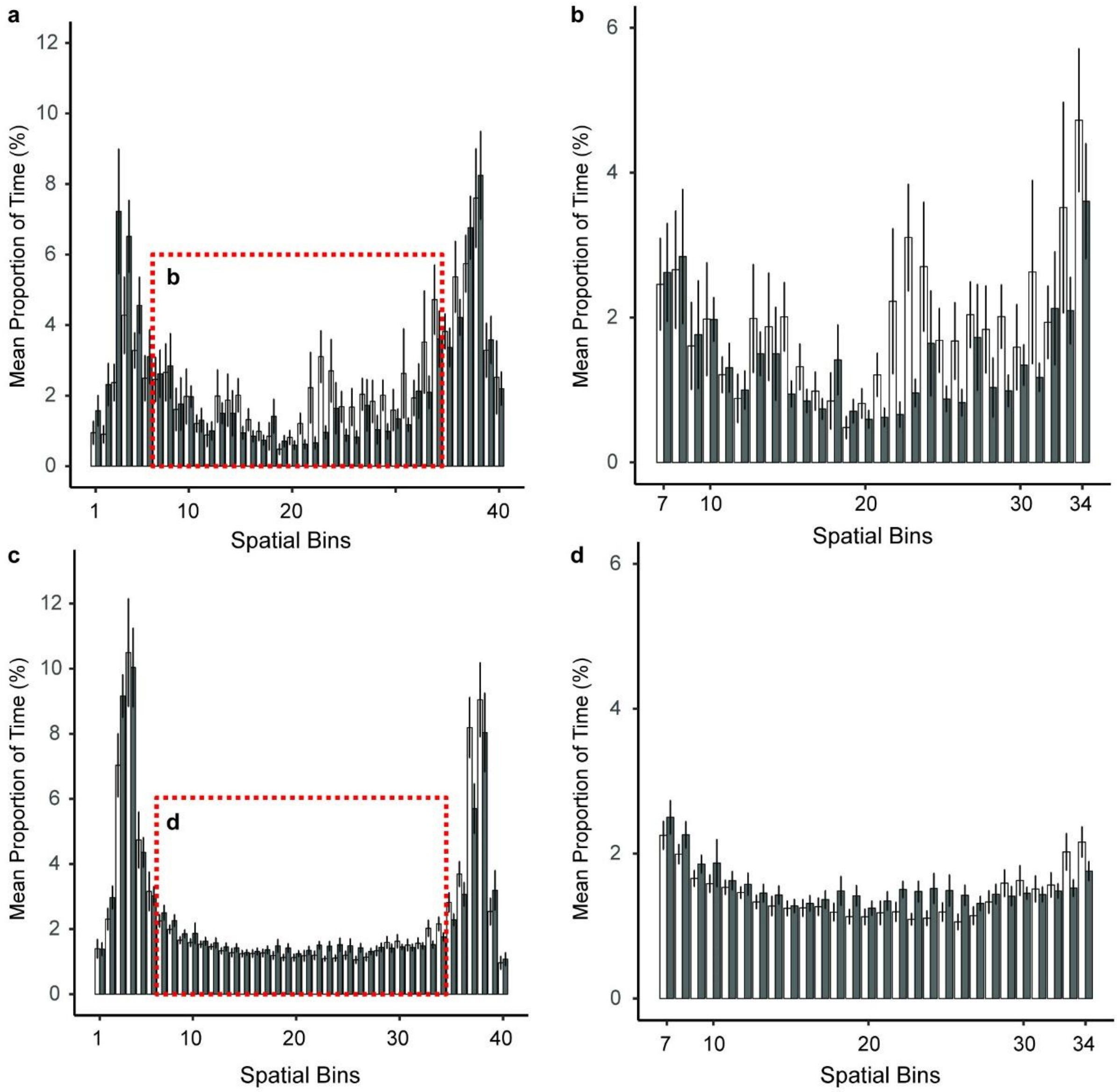

Figure S2. The spatial distribution pattern of skates and lobsters. The spatial distribution pattern of skates (a, b) and lobsters (c, d) shown as the mean (±SE) proportion of time (%) spent in each bin within the control (white) and treatment (grey) enclosures. The full length of the enclosure for skates and lobsters is shown (a, c) as well as the subset of data (b, d) focusing on the central area of the enclosures highlighted by the red box.

Section S3: Zones of EMF

The animal enclosure was positioned on top of the buried cable at a 94 degree angle, such that the cable crossed the base of the enclosure at an 86 degree angle and was off-centre. Using the data from the diver transects, the magnetic field was extrapolated in order to show the gradient of magnetic field that the animals were exposed to, at the base of the enclosure, when the cable operated at 330MW. Zones of ‘high’ and ‘low’ EMF (Figure S3) were defined based on the magnetic field when the cable was operating at full power (330 MW) and were based on two dimensions only (x, y). Zone 1 was defined as an area of high magnetic field, which ranged from 52.6 to 65.4 μT with a mean of 60.1 μT. Zone 2 was defined as an area of low magnetic field which ranged from 47.8 to 49.7 μT with a mean of 48.7 μT. The area of each zone differed; Zone 1 was 10.58 m2 while Zone 2 was 12.18 m^2. For the purposes of comparison, a correction factor was applied to behavioural parameters measured in Zone 2. Note that Zone 1 and Zone 2 were separated by a buffer of 30 cm to ensure no overlap. Zone 1 and Zone 2 were also spatially defined at the control enclosure for comparison but the magnetic field was constant throughout at 51.3 μT.

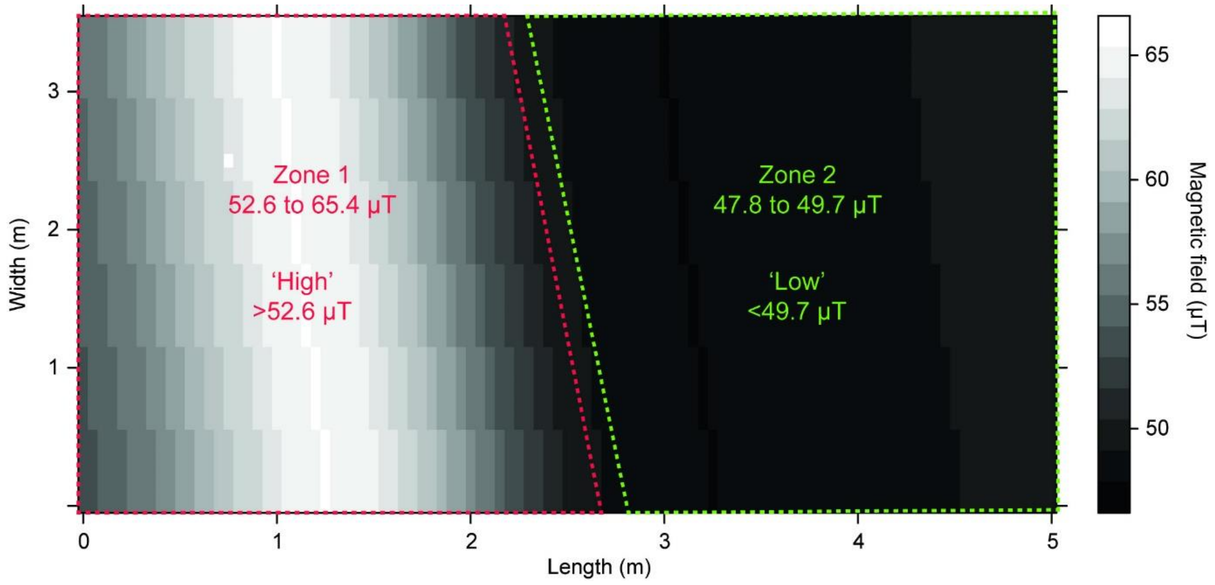

Figure S3. Zones of EMF. A top-down view of the base of the treatment enclosure showing the gradient of EMF at the seabed and the spatially defined zones of ‘high’ and ‘low’ EMF relative to the Earth’s magnetic field which was constant at 51.3 μT.

Section S4: Electromagnetic Field Modelling

COMSOL model development

The COMSOL model was first developed for the Cross Sound Cable at the location of the treatment enclosure and then adjusted to simulate the EMF from the Neptune cable. The EMF of the cable was simulated by defining the geometry (shape and size of the objects), the materials of each part of the object and the mesh of the whole object specified by the distribution and number of elements.

Geometric Simulation: Two HVDC cables (a bundled cable pair) are buried. The cables were modelled as a straight cylinder with infinite length and studied in a cross-section with a 2D model. The whole analysis domain is a circle and divided into two main parts; the upper and lower parts representing the sea and the seabed, respectively. The outer layer of the circle was set as the infinite domain. In the middle of the model, two cables were located in the seabed domain. The real layers of the cable (different materials with different functionality) were combined or omitted according to their electromagnetic properties. In the model, each cable is bundled by a lead sheath (electrostatic shield), filled with polyethylene XLPE (an insulator) and covered by a layer of steel armour (strength and protection). The geometric parameters of the model for the CSC were a burial depth of 1.5 m, a distance of 0.106 m between the two cables, a cable radius of 0.053 m, armour thickness of 0.01 m, lead sheath radius of 0.041 m and conductor radius of 0.235 m.

Materials: The CSC contains two HVDC cables that carry a pair of opposite-directed currents. In this simulation, the absolute value of each current was set to the maximum value of the transmission current, 1175 A. According to the Electromagnetic Induction Principle, each current will generate a stationary magnetic field. The two magnetic fields should cancel each other if the cables are perfectly overlapped. However, there is a distance between the two conductors resulting in a magnetic field. To simulate the EMF generated by the currents, the electromagnetic properties of the material are defined in Table S1. The permittivity ε (F/m) and the permeability μ (H/m) of each material are given in terms of their relative values ε_r and μ_r, respectively. The permittivity and permeability are derived by;

$$\varepsilon = \varepsilon_r \cdot \varepsilon_0$$

$$\mu = \mu_r \cdot \mu_0,$$

where ε_0 and μ_0 are the permittivity and the permeability of vacuum, and the values of them are $8.8542 \times 10^{-7}\mathrm{F/m}$ and $4\pi \times 10^{-7}\mathrm{H/m}$, respectively.

Table S1. Electromagnetic properties of materials for the cable models.

Layer	Electrical conductivity σ (s/m)	Relative permittivity ε_r	Relative permeability μ_r
Conductor (Copper)	5.8e7	1.0	1.0
Sheath (lead)	1e6	1.0	1.0
Insulator (XLPE)	0	2.3	1.0
Armour (Steel wire)	1.1e6	1.0	1000
Seawater	1.0	81.0	1.0
Seabed	0.25	25.0	1.0

Mesh: The Free Tetrahedral mesh was applied for the whole analysis domain. A quadrilateral mesh is used on the infinite domain. The cable dimension is much smaller in comparison to the whole model and the nearby EMF varies quickly therefore the mesh density around the cables was increased. The complete mesh of the model consists of 17952 domain elements and 1499 boundary elements.

Background magnetic field: Since the EMF generated by the cable will superimpose on the local geomagnetic field, geomagnetic information also needs to be considered. The local geomagnetic field at the enclosure location was estimated based on geomagnetic maps provided by the National Centers for Environmental Information[69]. The approximate local geomagnetic flux density distribution was incorporated including; the vertical component of 47 μT, north component of 20 μT and east component of -5 μT. A Cartesian coordinate system was built where the cable lay on the z-axis, the x-axis points to northeast, and the y-axis points to the vertical direction of the earth. In this local coordinate system, the y component of the geomagnetic field is -47 μT and the x and z component were calculated by vector decomposition to be 10.6 μT and -17.7 μT, respectively. Therefore, the corresponding local geomagnetic flux density could be written as $\left(B_x^b, B_y^b, B_z^b\right) = (10.6, -47, -17.7)\mu\mathrm{T}$, and the magnetic intensity of the background magnetic field is around 51.3 μT. The magnitude of the total magnetic field can be calculated by;

$$\|B_{tot}\| = \sqrt{(B_x^b + B_x)^2 + (B_y^b + B_y)^2 + (B_z^b)^2} \,.$$

Simulation: In COMSOL, the 2D AC/DC module describes the EMF with the following equations:

$$\nabla \times \mathrm{H} = \mathrm{J}$$

$$\mathrm{B} = \nabla \times \mathrm{A}$$

$$\mathrm{J} = \sigma\mathrm{E} + \sigma\mathrm{v} \times \mathrm{B} + \mathrm{J_e},$$

where H is the magnetic field intensity, J is the current density, B is the magnetic flux density, A is the magnetic vector potential, E is electric field intensity, v is the velocity of the conductor, and J_e is the externally generated current density. Among these variables, the magnetic vector potential A is the dependent variable. These equations will be solved with numerical iteration algorithm. For the model presented above, FGMRES (flexible generalised minimal residual method) was chosen as the solver and the relative error tolerance was set to 0.001. Moreover, the initial value of A was set to 0.

Neptune Cable: The EMF of the Neptune cable was simulated in the same manner with the following adaptations. The geometric parameters of the model for the Neptune cable were a burial depth of 1.4 m, a distance of 0.1155 m between the two cables, two cable radius of 0.063 m and 0.042 m, armour thickness of 0.01 m, lead sheath radius of 0.041 m for cable 1 and 0.03 m for cable 2 and lead sheath thickness of 0.04 m, and conductor radius of 0.0235 m. Considering the geographical position of the cable, the local vertical component is again, approximately -47 µT, the local North component is approximately 20 µT and the local east component is approximately -5 µT. In this coordinate system, the cable should be parallel to the z-axis and the corresponding local geomagnetic flux density could be written as $\left(B_x^b, B_y^b, B_z^b\right) = (-20, -47, -5)\mu T$. Thus, the magnitude of the background B-field is approximately 51.3 µT. The EMF was simulated based on an operational current of 1320 A.

COMSOL model simulations

The simulation did successfully converge (Figure S4a). Figure S4b provides a visualization of the magnetic field from the CSC; the arrow direction denotes the direction of the magnetic field and the arrow length is the logarithmic of the magnitude of the magnetic field. It is clear that the CSC is the source of the EMF. The total magnetic flux density distribution in the ocean domain for the CSC and NC is shown in Figure S4c & d. In both cases, the magnitude of the magnetic field decreases to a value that is close to that of the background magnetic field at an estimated distance of 8-10 m.

To show the modelled magnetic field for each cable at maximum capacity more clearly (CSC at 1175 A, NC at 1320 A), the magnitude of the total magnetic field along several parallel routes, corresponding to increasing increments of height above the seabed was plotted (Figure S4d & e). The first route is located on the boundary between the seabed and ocean (i.e. at 0 m) with increasing height increments of 0.5 m. The blue line is the total magnitude of the magnetic field on the seabed, which has peak values of approximately 66 μT for the CSC (Figure S4d) and 72 μT for the NC (Figure S4e). In both models, the magnitude of the magnetic field decreases with increasing 0.5 m steps from the seabed. The modelled magnetic field of the CSC is similar to the measured fields at different heights from the seabed, in the treatment enclosure (Figure 4b). Comparing the modelled magnetic fields of these two cables, it is clear that both NC and CSC can generate similar influence on the surrounding environment. The major distinction lays in the magnetic field magnitudes, which is mainly induced by the difference of the current intensities of these two cables.

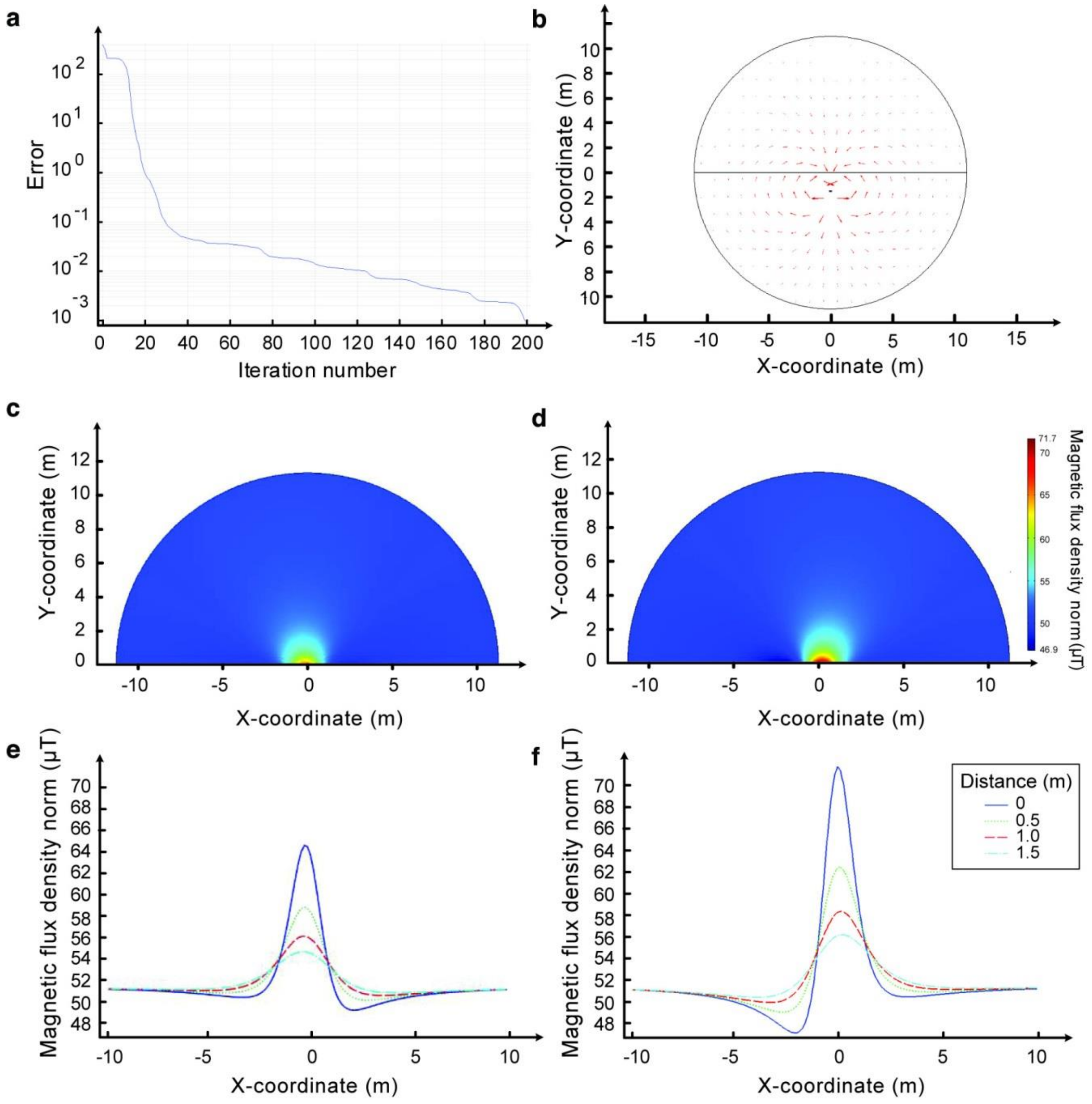

Figure S4. COMSOL simulations of the Cross Sound Cable (CSC) and Neptune Cable (NC). (a) Convergence process of the EMF simulation of the CSC. (b) Magnetic flux density in the analysis domain developed for the CSC. Total magnetic flux density distribution in the ocean for the CSC (c) and the Neptune Cable (d). The magnetic flux density at different heights from the seabed for the CSC (e) and the NC (f).

Fast model development

A fast numerical model (herein, 'fast model') of the two bundled cables was developed and used for estimating cable configuration at the enclosure. The model was employed since it can be iteratively used for predicting the optimal parameters, whereas a Finite Element Model such as the COMSOL model is too slow to be used in this application. The fast model estimated the magnetic DC-field generated by two bundled cables placed in a non-magnetic and non-conductive media at a specific height above the cable pair. In contrast to the

COMSOL model, the fast model did not account for the magnetic properties of the cable. The current was kept fixed at 1175 A but the angle between the cables relative to the vertical direction, the separation of cable centres and burial depth, were selected as free parameters. Only the measured magnetic fields at the seabed were used in the optimisation. The objective function, *L*, was defined as:

$$L = \sum_{n=1}^{20} \|B_{measured} - B_{modeled}\|^2$$

where *B* corresponds to magnetic flux density of the DC-field. The error per fitted point (*L/20*), was 0.28 $(\mu T)^2$.

The model predicted that the maximum magnetic DC-field at seabed was 65.3 μT, the field at mid-level was 55 μT and at the top of the enclosure even lower, 53.5 μT. Note that the model was used to derive the levels at mid and top levels, based on the fitted parameters from the optimization made on magnetic fields at the seabed. There was good agreement between the modelled and measured EMF at the site of the treatment enclosure (Figure 4b).

Section S5: Supporting Figure

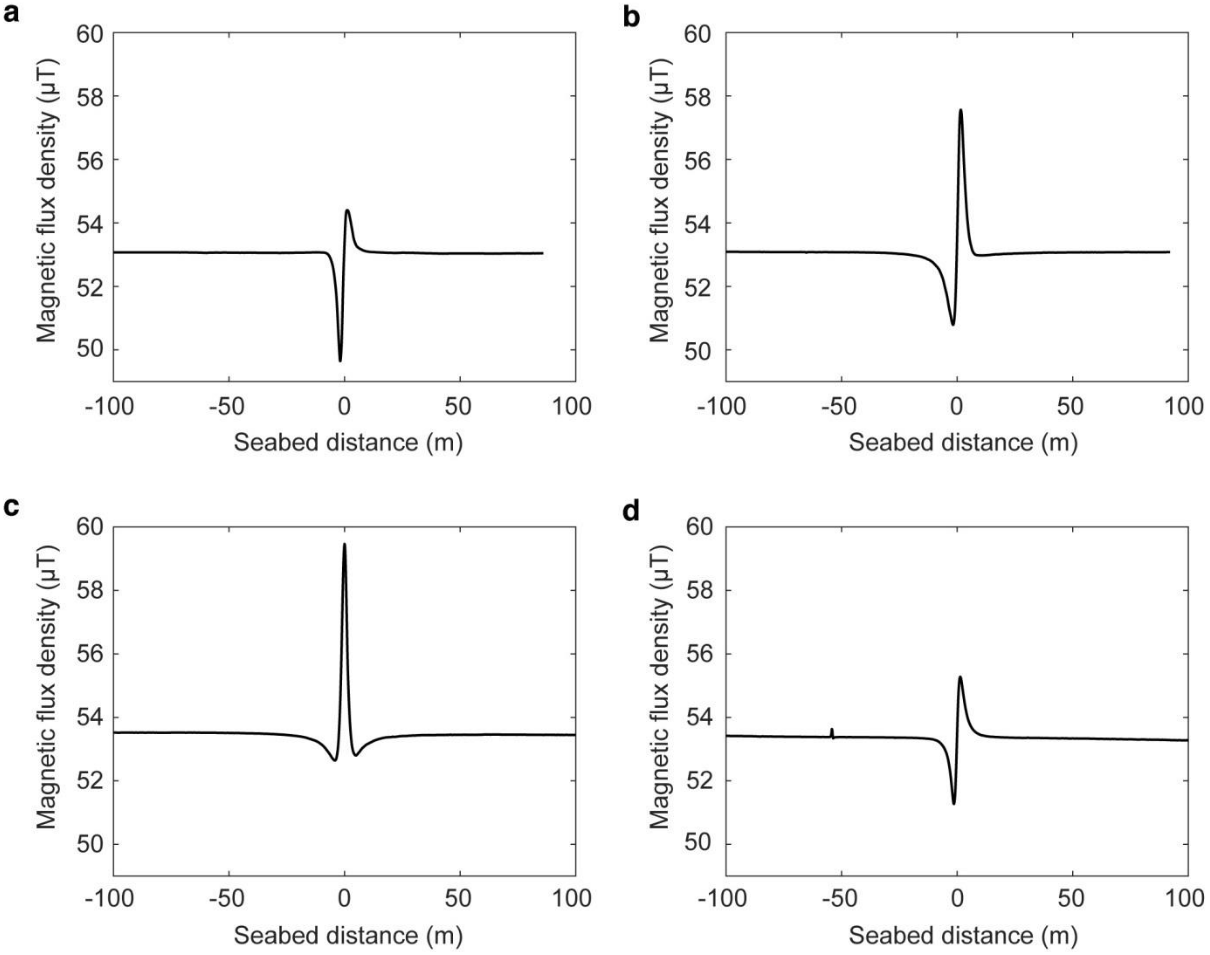

Figure S5. Spatial extent and variation in the symmetry of the total magnetic DC-field. The shape of the magnetic field emitted from the cable is influenced by the orientation of the two internal cables to each other. The two internal cables were rotated relative to the vertical direction, which accounts for the variable symmetry of the magnetic field observed. This is demonstrated by four transects of the NC: three at 1320 A (a, b, c) and one at 660 A (d).

Section S6: Statistical Analyses

Data plots were produced using 'ggplot2'[70]. Data exploration was conducted following the protocol outlined by Zuur et al., 2010[71].

Spatial distribution: The 3D positions were used to determine the spatial distribution within the enclosures. The patterns of distribution, assessed by the proportion of time spent in 40 spatial bins (c.a. 14 cm each) of the length of the enclosure. Comparisons between enclosures were made by a non-parametric Kolmogorov-Smirnov two sample test for the full length of the enclosure (i.e. bins 1-40) and the central space of the enclosure (i.e. bins 7-34).

Behavioural parameters: The total distance travelled per day, the speed of movement, the proportion of large turns (using the 'adehabitatLT' package[72]) and the height from the base of the enclosure (herein 'height from seabed') were calculated. The total distance travelled, the mean speed of movement and height from the seabed were fitted with a Gaussian distribution. Due to the repeated measures nature of the data, linear mixed effect models were used which allowed a fixed and random structure to be incorporated[68]. Response variables (the behavioural parameters) were log transformed where necessary. In the maximal model, the fixed effects were specified as the 'Enclosure' and the 'Sequence' plus the interaction between the two variables. Models with and without random structures were fitted using generalised least squares (gls) and linear mixed effect (lme) models in the 'nlme' package[73] using restricted maximum likelihood (REML) estimation and maximum likelihood (ML) as appropriate[68]. The proportion of large turns was assessed using a generalised linear mixed model using Penalized Quasi-likelihood (glmmPQL) with a binomial distribution (bound between 0 and 1) using the 'MASS' package[74]. Validation of the model was based on plots of the fitted values and the Pearson residuals to check that the model assumptions were met. The random structure must be specified in a glmmPQL so in order to explore a model without a random structure a generalised linear model (glm) was also explored, fitted with binomial distribution and quasi-correction where appropriate. Model selection for the glm (i.e. simplification) was based on the Akaike Information Criterion (AIC)[75], using the 'drop1' function with chi-squared/F tests as appropriate. Comparisons between glmmPQL and binomial glm models were based on the validation plots only.

Zone comparison: To further assess the influence of the EMF on the behavioural parameters, the space in the enclosures were split into two zones; above or below the Earth's magnetic field, based on the EMF at the base of the enclosure (seabed). Zone 1 was 'high'

(52.6 to 65.4 μT, 10.58m^2) and Zone 2, low (47.8 to 49.7 μT, 12.18 m^2). A correction factor accounted for the areal difference and zones were separated by 30 cm buffer to ensure no overlap. The zones were defined based on the magnetic field at the treatment enclosure and the same spatial zones were defined at the control enclosure (constant 51.3 μT) for comparison. Only the behavioural parameters which showed significant differences between enclosures were assessed in this way. For each of the behavioural parameters of interest, the differences between zones were assessed and the group means compared between control and treatment enclosures using a Welch's two sample t-test.
